# HER2+ breast cancer treatment and cardiotoxicity: monitoring and management

**DOI:** 10.1007/s10549-019-05303-y

**Published:** 2019-06-05

**Authors:** Guy Jerusalem, Patrizio Lancellotti, Sung-Bae Kim

**Affiliations:** 1 Department of Medical Oncology, Centre Hospitalier Universitaire du Sart-Tilman, University of Liege, Liège, Belgium; 2 GIGA Cardiovascular Sciences, Department of Cardiology, Centre Hospitalier Universitaire du Sart-Tilman, University of Liege, Liège, Belgium; 3 Asan Medical Center, University of Ulsan College of Medicine, 88, Olympic-ro 43-gil, Songpa-gu, Seoul, 05505 Korea

**Keywords:** Breast neoplasms, Cardiotoxicity, Trastuzumab, Anthracyclines

## Abstract

**Background:**

Breast cancer is a leading cause of death for women worldwide, with incidence increasing in lower-income countries. For patients with human epidermal growth factor receptor-2-positive (HER2+) breast cancer, widespread availability of several agents targeting the HER2 receptor has resulted in survival gains over the past decades. However, improved survival has resulted in an increased need for management and mitigation of adverse events associated with anticancer therapy. Cardiac adverse events such as decreased ejection fraction and heart failure have been of particular concern in patients with HER2+ breast cancer. Anti-HER2 agents and chemotherapies (specifically anthracyclines, which are frequently used to treat HER2+ disease) have been associated with cardiotoxicity. As increasing numbers of patients are living longer due to more effective therapy, a better understanding of both monitoring and management of cardiotoxicity is urgently needed.

**Methods:**

A comprehensive review of the literature was conducted via PubMed in January 2018 for phase II and phase III trials of “trastuzumab”, “lapatinib”, “pertuzumab”, “T-DM1”, “neratinib”, in “breast cancer”. Literature was evaluated for content related to cardiac adverse events.

**Findings:**

We describe the incidence of and proposed mechanisms for the cardiotoxicity of available HER2-targeted therapies. We summarize current and emerging practices in the management of cardiotoxicity and provide guidance for routine patient care in real-world practice using illustrative patient scenarios.

**Conclusions:**

The future of cardiotoxicity management in patients with HER2+ breast cancer is discussed, with a focus on novel techniques to improve cardiac outcomes, including new imaging modalities, biomarkers, interventional therapies, and ongoing trials.

## Introduction

Human epidermal growth factor receptor-2 (HER2+) breast cancers compose approximately 15% of all breast cancers and are more likely to be diagnosed in younger patients and at a more advanced stage than the more common hormone receptor-positive, HER2-negative breast cancers [1]. However, treatments that specifically target the HER2 receptor have significantly improved survival rates for patients with both early- and advanced-stage disease [2]. Currently, several anti-HER2 therapies have been approved for the treatment of advanced and/or early breast cancer and additional agents are in late-stage clinical development (Table 1) [3–15].

**Table 1:** Key trials in targeted therapies for HER2+ breast cancer

Agent(s)	Treatment	Setting	Patient number	Results	References
Trastuzumab	Phase 3 trial of CT + TRAS vs CT alone	ABC; no prior CT for advanced disease	469	Significantly improved time to progression and OS	Slamon et al. [3]
Lapatinib	Phase 3 trial of LAP + CAP vs CAP alone	ABC with progression on TRAS + CT	324	Significantly improved time to progression	Geyer et al. [4]
	Phase 3 trial of LAP + LET vs LET alone	HR+, HER2+ ABC with no prior treatment for advanced disease	219	Significantly improved time to progression	Johnston et al. [5]
Lapatinib + trastuzumab	Phase 3 trial of LAP + TRAS vs LAP alone	MBC with progression on TRAS-based therapy	296	Significantly improved time to progression and OS	Blackwell et al. [6]
	Phase 3 trial of TRAS vs LAP vs TRAS + LAP (NeoALTTO)	Neoadjuvant therapy for HER2+ breast cancer	455	Significantly improved pCR for the LAP + TRAS arm	Baselga et al. [7]
	Phase 3 trial of TRAS vs LAP vs TRAS → LAP vs TRAS + LAP (ALTTO)	Initial adjuvant therapy for HER2+ EBC	8381	No significant difference in DFS across arms	Piccart-Gebhart et al. [8]
Pertuzumab + trastuzumab	Phase 3 trial of pertuzumab + TRAS + CT vs TRAS + CT (CLEOPATRA)	HER2+ ABC with no prior treatment for advanced disease	808	Significantly improved time to progression and OS	Swain et al. [9]
	Phase 3 trial of pertuzumab + TRAS + CT vs TRAS + CT (APHINITY)	Initial adjuvant therapy for HER2+ EBC	4805	Significantly improved iDFS	Von Minckwitz et al. [10]
	Phase 2 study of pertuzumab ± TRAS, TRAS + CT, or pertuzumab + CT (NeoSphere)	Treatment-naive early or locally advanced HER2+ BC	417	Dual targeting group had significantly improved complete response rate	Gianni et al. [11]
Trastuzumab emtansine (T-DM1)	Phase 3 study of T-DM1 vs LAP + CAP (EMILIA)	ABC with progression on TRAS + CT	991	T-DM1 significantly prolonged PFS and OS	Verma et al. [12]
	Phase 3 study of T-DM1 vs physician’s treatment of choice (TH3RESA)	ABC with previous treatment including TRAS, LAP, and a taxane	602	T-DM1 significantly prolonged OS	Krop et al. [13]
Neratinib	Phase 2 study of neratinib + paclitaxel vs TRAS + paclitaxel (NEfERT-T)	Previously untreated HER2+ MBC	479	Non-superior efficacy of neratinib vs TRAS	Awada et al. [14]
	Phase 3 trial of neratinib vs placebo as extended adjuvant therapy (ExteNET)	HER2+ EBC who completed (neo)adjuvant TRAS + CT	2840	Significantly reduced risk of clinically relevant BC relapse	Martin et al. [15]

*ABC* advanced breast cancer, *BC* breast cancer, *CAP* capecitabine, *CT* chemotherapy, *EBC* early breast cancer, *DFS* disease-free survival, *HER2+* human epidermal growth factor receptor-2-positive, *HR+* hormone receptor-positive, *iDFS* invasive disease-free survival, *LAP* lapatinib, *LET* letrozole, *MBC* metastatic breast cancer, *OS* overall survival, *pCR* pathological complete response, *PFS* progression-free survival, *T-DM1* trastuzumab emtansine, *TRAS* trastuzumab

Current guidelines for the management of HER2+ disease focus on optimal disease control with limited toxicity by combining sequential HER2-targeted therapy with chemotherapy [16–19]. As treatment efficacy increases, there are increasing numbers of patients who survive for extended periods and may receive therapy for a prolonged duration. Therefore, patients increasingly require long-term management of treatment morbidities. Cardiac health is an issue of special concern for HER2+ breast cancer as both chemotherapy and HER2-targeted therapies can cause cardiotoxicity [20, 21]. Better awareness of the late effects of treatment-related cardiotoxicity and strategies for long-term management are urgently needed. Here, we review the cardiotoxicity risks of HER2-targeted therapies and the currently recommended clinical guidelines for monitoring and management. We further summarize emerging techniques for the identification, treatment, and monitoring of patients with increased risk of cardiotoxicity.

## Cardiotoxicity of currently available HER2-targeted therapies

**Fig. 1:**
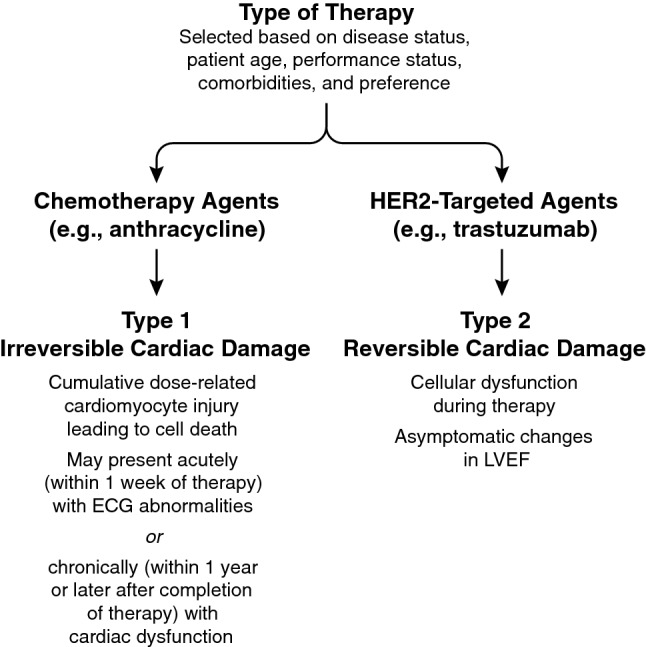
Schematic of Potential Mechanisms of Cardiotoxicity. There are 2 types of therapy selected based on disease status, patient age, performance status, comorbidities, and preference. Chemotherapy agents such as anthracycline can lead to irreversible (type 1) cardiac damage. With type 1 damage, cumulative dose-related cardiomyocyte injury leading to cell death can occur and may present acutely (within 1 week of therapy) with ECG abnormalities or chronically (within 1 year or later after completion of therapy) with cardiac dysfunction. HER2-targeted agents such as trastuzumab can lead to reversible (type 2) damage. With type 2 damage, cellular dysfunction during therapy and asymptomatic changes in LVEF can occur. *ECG* electrocardiogram, *HER2* human epidermal growth factor receptor-2, *LVEF* left ventricular ejection fraction

Anticancer agents in routine clinical use may cause cardiac damage via several distinct mechanisms. This may include irreversible damage (Type 1; e.g., with chemotherapeutic agents such as anthracyclines), or reversible dysfunction (Type 2; e.g., with HER2-targeted therapies) [22] (Fig. 1). Although many therapies carry a risk of cardiotoxicity, types and rates of cardiac adverse events (AEs) reported in the literature vary widely (Table 2) [3–5, 7–10, 12, 14, 15, 21, 23–29]. As increasing numbers of patients are being treated with HER2-targeted therapies for extended periods of time, frequently with multiple agents and/or in combination with anthracycline-based chemotherapy, it is essential to fully understand these mechanisms to implement appropriate clinical monitoring and management.

**Table 2:** Cardiac adverse events in clinical trials of HER2-targeted therapy

Agent(s)	Outcome measure	Cardiac safety data	References
Trastuzumab	Cardiac dysfunction (symptomatic or asymptomatic) for patients receiving TRAS + CT vs CT alone	• 27% of patients receiving TRAS + anthracycline + cyclophosphamide vs 8% receiving anthracycline + cyclophosphamide 13% receiving TRAS + paclitaxel vs 1% receiving paclitaxel alone	Slamon et al. [3]
	Cardiac dysfunction noted in retrospective analysis of phase 2–3 clinical trials (*N* = 202)	• 27% of patients receiving TRAS + anthracycline + cyclophosphamide vs 13% of patients receiving TRAS + paclitaxel vs 3–7% receiving TRAS alone • Majority of patients with TRAS-related cardiotoxicity (75%) were symptomatic	Seidman et al. [23]
	Meta-analysis of patients receiving TRAS for metastatic breast cancer (*N* = 1497)	• Significant increase of CHF for patients receiving TRAS (RR = 3.49; 90% CI 1.88–6.47; *P* = .0009) • Significant increase of LVEF decline for patients receiving TRAS (RR = 2.65; 90% CI 1.48–4.74; *P* = .006)	Balduzzi et al. [24]
	Meta-analysis of patients receiving adjuvant TRAS in clinical trials (*N* = 18,111)	• Significant increase of high-grade CHF for patients receiving TRAS (RR = 3.19; 95% CI 2.03–5.02; *P* < .00001)	Long et al. [21]
Lapatinib	Cardiac events for patients receiving LAP + CAP vs CAP alone	• Asymptomatic cardiac events in four patients receiving LAP + CAP vs one patient receiving CAP alone • No symptomatic events and no difference in mean LVEF values between groups	Geyer et al. [4]
	LVEF decline for patients receiving LAP + LET vs LET alone	• Symptomatic LVEF decline in five patients receiving LAP + LET vs two patients receiving LET alone	Johnston et al. [5]
	Meta-analysis of patients receiving LAP in clinical trials (*N* = 3689)	• Study-defined cardiac events were reported in 1.6% of patients, were generally asymptomatic, and occurred at similar rates regardless of prior treatment	Perez et al. [25]
Lapatinib + trastuzumab	Phase 3 trial of LAP + TRAS vs LAP alone in patients with disease progression on TRAS	• 11 patients in the combination arm vs 3 patients in the monotherapy arm experienced cardiac events • Ten events in the combination arm were serious events, including one death	Blackwell et al. [26]
	NeoALTTO clinical trial	• A single patient in each treatment arm experienced decreased LVEF • One patient receiving LAP + TRAS experienced class III CHF (recovered after treatment interruption)	Baselga et al. [7]
	ALTTO clinical trial	• Low incidence of primary cardiac events (0.25–0.97% of patients)	Piccart-Gebhart et al. [8]
Pertuzumab + trastuzumab	CLEOPATRA clinical trial	• 27 patients (6.6%) in the PERT group vs 34 patients (8.6%) in the placebo group had reduced LVEF over the course of the study	Swain et al. [9]
	APHINITY clinical trial	• 17 patients (0.7%) in the PERT group vs 8 patients (0.3%) in the placebo group experienced a primary cardiac event	Von Minckwitz et al. [10]
Trastuzumab emtansine (T-DM1)	EMILIA clinical trial	• LVEF decrease of ≥ 15% from baseline in 1.7% of patients treated with T-DM1 vs 1.6% treated with LAP + CAP	Verma et al. [12]
	TH3RESA clinical trial	• LVEF decrease of ≥ 15% from baseline in 1% of patients treated with T-DM1 vs 1% treated with physician’s choice of therapy	Krop et al. [27]
	Phase 2 adjuvant setting	• No protocol-specified cardiac safety or CHF events for patients receiving T-DM1	Krop et al. [28]
	MARIANNE clinical trial	• LVEF decrease of ≥ 15 points from baseline in 0.8% of patients treated with T-DM1 vs 4.5% treated with TRAS + taxane vs 2.5% T-DM1 + PERT	Perez et al. [29]
Neratinib	NEfERT-T clinical trial	• Grade 3 + cardiac adverse events were reported in three patients (1.3%) receiving neratinib + paclitaxel vs seven patients (3.0%) receiving TRAS + paclitaxel	Awada et al. [14]
	ExteNET	• Noted no evidence of increased long-term symptomatic cardiac safety (specifics not reported)	Martin et al. [15]

*CAP* capecitabine, *CHF* congestive heart failure, *CI* confidence interval, *CT* chemotherapy, *HER2* human epidermal growth factor receptor-2, *LAP* lapatinib, *LET* letrozole, *LVEF* left ventricular ejection fraction, *PERT* pertuzumab, *RR* response rate, *TRAS* trastuzumab, *T-DM1* trastuzumab emtansine

### Trastuzumab

Trastuzumab is frequently associated with an increased risk of cardiotoxicity, with 3% to 7% of patients who received trastuzumab monotherapy in clinical trials experiencing some form of cardiac dysfunction [23]. Although the mechanism for trastuzumab-associated cardiac dysfunction is not fully understood, it has been suggested that cardiomyocyte death occurs via multiple pathways, including as the direct result of ErbB2 (HER2) blockade and the increased production of reactive oxygen species [30, 31].

Administration of trastuzumab after prior treatment with anthracyclines may contribute to the relatively high levels of cardiotoxicity reported in clinical trials [32]. Although up to 75% of patients in clinical trials of trastuzumab who exhibited cardiac dysfunction were symptomatic, the majority of these had prior treatment with anthracyclines [23]. In contrast, there is a low incidence of symptomatic heart failure and decline in asymptomatic left ventricular ejection fraction (LVEF) among patients receiving trastuzumab without an anthracycline [23, 32]. In addition to the risks of trastuzumab administered with chemotherapy, concomitant radiotherapy to the left breast (or left thoracic wall) may be associated with an increase in long-term cardiotoxicity risk, although this phenomenon is not yet fully understood [33]. More research is needed to better understand the potential cardiac risks of trastuzumab, and multimodal therapies frequently used to treat aggressive disease.

Both clinical trial data and real-world evidence suggest an increased risk of cardiotoxicity with trastuzumab treatment. In the initial phase 3 trial of trastuzumab in patients with HER2+ metastatic breast cancer (MBC), 27% of patients receiving trastuzumab plus chemotherapy experienced cardiac dysfunction, compared with 8% of patients receiving chemotherapy alone [3]. Across seven trials in patients with MBC (*N* = 1497), the risk of cardiac toxicities was significantly increased in patients receiving trastuzumab-containing regimens compared with regimens that did not contain trastuzumab, even though normal cardiac function was an entry criterion for all but the initial trial [24]. Risks of both serious cardiac dysfunction [including congestive heart failure (CHF)] and LVEF decline were significantly increased in patients receiving trastuzumab, irrespective of whether it was administered as a first-line treatment or beyond disease progression [24]. In a real-world cohort of patients receiving trastuzumab for primary or MBC (*N* = 388), cardiotoxicity occurred at a higher-than-expected rate and occurred more frequently in patients who were older and had prior treatment with an anthracycline [34]. These data confirm the need for cardiac monitoring throughout treatment, particularly for patients with MBC, who are more likely to be older and to have received prior anticancer therapy.

In the adjuvant setting, long-term safety and late-onset AEs are issues of special concern because patients may receive treatment for an extended duration. A meta-analysis study reported increased risk of asymptomatic LVEF decrease and symptomatic heart failure with adjuvant trastuzumab [24]. Although overall incidence is low, adjuvant trastuzumab has been associated with a significant increase in risk of serious cardiac events such as CHF [21]. Cardiotoxicity remains an issue of special concern when trastuzumab is combined with an anthracycline in the adjuvant setting even though therapies are administered sequentially [35]. A Dutch study of patients receiving trastuzumab-based adjuvant therapy in a real-world setting (*N* = 230) reported cardiotoxicity in 12.6% of patients, with 8.7% of these patients experiencing symptomatic cardiotoxicity [36]. An Italian study of women receiving trastuzumab for early breast cancer (*N* = 499) noted even higher rates of cardiotoxicity, with 27% of patients experiencing LVEF decrease [37]. Importantly, both studies note that trastuzumab-related cardiotoxicity appears early in the course of treatment (≤ 6 months of therapy), highlighting the crucial need for early monitoring [36, 37]. Indeed, the trastuzumab label includes guidance to monitor cardiac function before starting treatment, at regular intervals throughout the course of therapy, and every 6 months for at least 2 years after completion of adjuvant therapy [38, 39].

### Lapatinib

Lapatinib, an epidermal growth factor receptor (EGFR) and HER2 dual tyrosine kinase inhibitor (TKI), is infrequently associated with cardiac AEs such as decreased LVEF in clinical trials [4, 5, 29]. A meta-analysis demonstrated that the majority of patients treated with lapatinib in clinical trials who had decreased LVEF was asymptomatic and that the effect was largely reversible with treatment interruption [29]. It is important to note that the majority of patients in clinical trials of lapatinib was previously treated with trastuzumab but was excluded from these trials if prior cardiac toxicity was observed. Although a meta-analysis of data for patients receiving lapatinib in clinical studies demonstrated similar rates of LVEF decline with or without trastuzumab or anthracycline pretreatment [29], the ALLTO trial allowed a head-to-head comparison between trastuzumab and lapatinib and demonstrated fewer cardiac events (including CHF and LVEF decrease) with lapatinib versus trastuzumab at 1 year [8]. As lapatinib is frequently administered to patients who have previously received therapy with trastuzumab and/or anthracycline-based chemotherapy, label guidance indicates LVEF monitoring at baseline and during treatment [40, 41].

The possibility of dual targeting with lapatinib and trastuzumab was explored in several large phase 3 clinical trials. In the neoadjuvant setting, cardiac AEs occurred at a low rate in patients receiving both trastuzumab and lapatinib with paclitaxel (one patient in each group experienced LVEF decrease) [7]. In contrast, in the adjuvant setting there was a higher incidence of AEs in patients receiving both lapatinib and trastuzumab, including cardiac endpoints (LVEF decrease or CHF) [8]. In the metastatic setting, the combination of lapatinib and trastuzumab resulted in a low incidence of cardiac events across treatment arms, which was notable as all patients had received prior lines of therapy that included both trastuzumab and an anthracycline [6]. Although duration of exposure to anti-HER2 therapies may have been shorter in the metastatic setting trials than in the adjuvant setting, these data overall indicate that the addition of lapatinib to trastuzumab does not drastically increase the risk of cardiac events over that associated with trastuzumab alone.

### Pertuzumab

As pertuzumab is administered in combination with trastuzumab for both early-stage and metastatic breast cancer, cardiotoxicity has been evaluated in several clinical trials. For patients receiving pertuzumab plus trastuzumab and docetaxel for HER2+ metastatic breast cancer in the phase 3 CLEOPATRA clinical trial, the long-term rate of cardiotoxicity was slightly lower than for patients receiving placebo plus trastuzumab and docetaxel [9]. Similarly, patients receiving pertuzumab in addition to standard adjuvant chemotherapy and 1 year of trastuzumab for early breast cancer in the APHINITY trial experienced low rates of cardiac events [10]. For patients receiving pertuzumab in the neoadjuvant setting, the phase 2 NeoSphere study reported a low incidence of cardiac events across treatment arms [11]. Finally, an analysis of 57 patients receiving pertuzumab-based combination therapy in a real-world setting did not note an increased risk of cardiotoxicity compared with sequential chemotherapy and trastuzumab [42]. However, as the available cardiac safety data are limited and as pertuzumab is often administered with anthracyclines and/or trastuzumab, the label carries a warning for LVEF decrease [43, 44].

### Trastuzumab emtansine (T-DM1)

T-DM1 is indicated for use as a single agent for metastatic HER2+ cancer after failure of trastuzumab [45]. The phase 3 TH3RESA and EMILIA clinical trials in previously treated HER2+ metastatic breast cancer noted similarly low rates of LVEF decrease for patients receiving T-DM1 or an alternate therapy (including trastuzumab or lapatinib plus chemotherapy or chemotherapy alone) [12, 27]. Initial clinical trials in the adjuvant setting noted that rates of LVEF decrease were lower than those observed in trials of trastuzumab plus chemotherapy, even after prior treatment with anthracyclines [46]. In the phase 3 MARIANNE trial, fewer patients treated with T-DM1 experienced LVEF decrease compared with those treated with trastuzumab plus a taxane [29]. The low rate of cardiac events was confirmed in a retrospective analysis of 250 patients receiving T-DM1 for metastatic disease in a real-world setting [47]. Although rates for cardiac AEs with T-DM1 were generally low, the drug label includes a warning for cardiotoxicity due to LVEF decrease, which may be of particular concern because the drug is indicated for patients with prior treatment including both trastuzumab and a taxane [45, 48].

### Neratinib

Neratinib, a TKI approved in 2017 for treatment of HER2+ breast cancer, was not associated with grade 3/4 cardiotoxicity for patients in a phase 2 trial, and little variation in LVEF versus baseline was noted across patients regardless of prior trastuzumab treatment [49]. Cardiotoxicity in phase 3 trials (ExteNET and NEfERT-T) was minimal and follow-up continues for monitoring of cardiac AEs [14, 50]. No evidence of increased long-term toxicity, specifically symptomatic cardiotoxicity, was observed in patients receiving neratinib in the adjuvant setting [15]. Neratinib is approved for use in the adjuvant setting following trastuzumab-based therapy and currently does not carry a warning for cardiotoxicity [51].

## Clinical monitoring and management of cardiotoxicity

### Current approaches

Guidelines for the monitoring and management of treatment-induced cardiotoxicity are available from the European Society for Medical Oncology (ESMO), the National Comprehensive Cancer Network (NCCN), the American Society of Clinical Oncology (ASCO), and the European Society of Cardiology (ESC) [17, 19, 52, 53]. When creating a treatment plan the individual risk factors for each patient must be carefully considered to choose both the appropriate therapy and the necessary cardiac monitoring plan. Although ongoing monitoring is recommended for patients receiving trastuzumab therapy, all guidelines note that the optimal interval has not been determined. As cardiotoxicity due to HER2-targeted therapy is well recognized, patients at increased risk for cardiac events are frequently excluded from trials, leaving little evidence for the treatment of patients with baseline cardiac disease, a common scenario in clinical practice. This underscores the need for studies such as the ongoing SAFE-HEaRT trial in patients with mild reductions in LVEF [54].

Interruption or cessation of HER2-targeted therapy is recommended if significant LVEF decrease is detected during treatment. However, several retrospective analyses of patients receiving trastuzumab in a real-world setting have noted LVEF monitoring is not generally performed as recommended in current guidelines [55, 56]. The discordance between clinical guidelines and real-world practice is further confounded by the fact that although serial LVEF monitoring during trastuzumab treatment is recommended, some debate exists as to the optimal frequency and utility of LVEF changes in predicting heart failure or in deciding on a change in treatment [57, 58]. Although the trastuzumab label recommends monitoring at baseline and every 3 months of treatment (with further monitoring recommended for patients who receive therapy in the adjuvant setting), the cost of this testing and the relatively low rate of patients requiring therapeutic intervention for symptomatic cardiac events keep this issue as a topic of debate [38, 59]. In particular, young patients with no history of cardiac disease may not derive significant benefit from cardiac monitoring and long-term follow-up [60].

Detection of clinically significant cardiac dysfunction before or during treatment may result in additional interventions beyond change of treatment plan. In patients with a high risk of cardiac dysfunction receiving anthracycline chemotherapy, dexrazoxane may be administered to reduce the risk of cardiotoxicity [61]. Although current evidence is limited, several studies suggest that standard treatments for cancer therapy-induced LVEF decrease and heart failure, such as angiotensin-converting-enzyme (ACE) inhibitors or beta-blockers, may preserve cardiac function in patients receiving trastuzumab or chemotherapy [62, 63]. Trials evaluating preventive treatments, such as the SAFE trial, are ongoing, with results eagerly anticipated [64].

### Considerations for special populations

Selection of treatment for patients with HER2+ breast cancer in either the adjuvant or advanced setting must balance potential benefits of treatment with risk for AEs. When specifically assessing the risk of cardiotoxicity, certain populations are at an elevated risk and pose a further challenge to the clinician in selecting an ideal treatment. These populations may have been excluded from (or under-represented in) clinical trials and require further evaluation. To illustrate the complex decisions that may face physicians, we describe 2 hypothetical case studies demonstrating common clinical scenarios.

#### Hypothetical case study 1

Despite the fact that cancer incidence increases with age, elderly patients are less frequently included in clinical trials, more frequently have comorbidities, and may be more likely to experience AEs [65]. Elderly patients are at a greater risk of developing cardiotoxicity due to cancer therapy, frequently due to an increased prevalence of underlying cardiac risk factors and common comorbidities such as diabetes and hypertension [53, 66]. However, cardiotoxicity data on elderly patients receiving HER2-targeted therapy are limited. A meta-analysis in patients receiving trastuzumab for HER2+ breast cancer in routine clinical practice found that elderly patients had a significantly increased risk of cardiac toxicity compared with younger patients [67]. A similar retrospective analysis of Medicare data noted that elderly patients receiving trastuzumab and/or chemotherapy experienced CHF at a higher rate than that observed in clinical trials, and that the risk increased with advancing age [68].

**Fig. 2:**
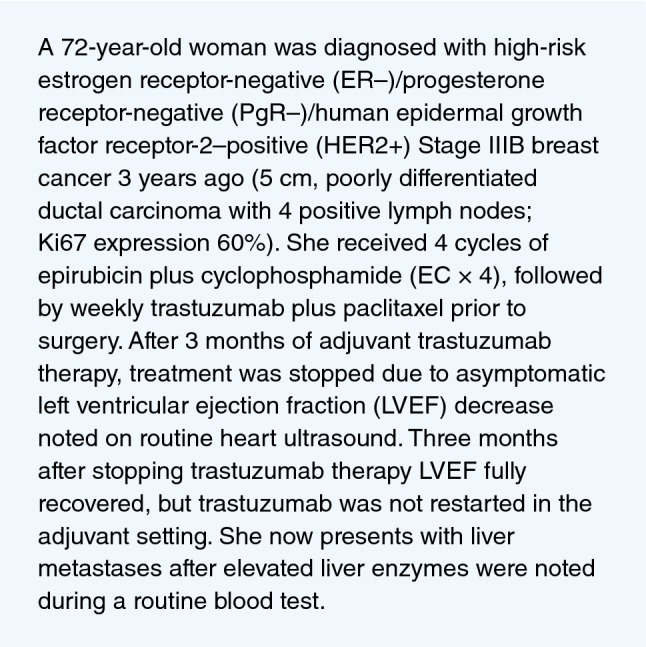
Hypothetical case study 1

In this case, the patient has experienced disease recurrence after receiving prior trastuzumab therapy (Fig. 2). Given her history of cardiotoxicity, precaution must be taken when deciding on a course of treatment including targeted therapy for metastatic disease. Based on the patient’s age, treatment history, and potential comorbidities, careful consultation with a cardiac oncology team should be sought before beginning treatment.

#### Hypothetical case study 2

Heart disease and additional comorbidities that may increase risk of cardiovascular disease (such as diabetes) are increasingly common [69]. The presence of chronic disease (including atrial fibrillation, ischemic heart disease, poorly controlled hypertension, and diabetes) can complicate cancer treatment [70]. The lack of prospective clinical trial data on patients with pre-existing cardiac disease further confounds the ability of physicians to select the appropriate anticancer therapy while appropriately continuing treatment for cardiac disease.

**Fig. 3:**
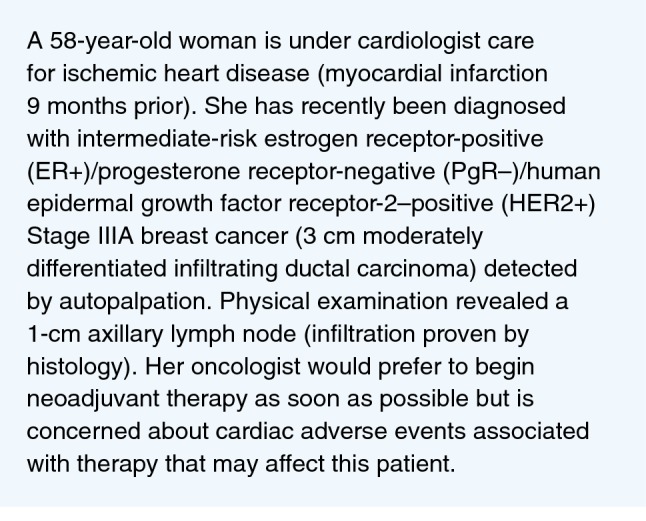
Hypothetical case study 2

In this case, the patient is already under a cardiologist’s care, so her baseline disease status and associated risk of AEs are clearly established (Fig. 3). However, many patients may not have received prior medical treatment, or may have subclinical cardiac disease that is unknown to them. Full assessment of cardiac function for each patient should take place before the start of treatment, and careful monitoring should be followed to reduce risk [71]. For patients with notable cardiac disease at baseline, early integration of a cardiologist or cardio-oncology team into treatment planning is essential for effective and safe disease management. Regardless of age or baseline status, every effort should be made to provide patients with an appropriate level of care to manage their breast cancer and concomitant cardiac health conditions.

## The future of monitoring and treatment for HER2-therapy-associated cardiotoxicity

With more patients living longer during and after cancer treatment, there is a need for earlier and more accurate detection of cardiotoxicity. Cardiac function in clinical trials and retrospective analyses is generally analyzed using LVEF changes, with symptomatic CHF as an endpoint. Although LVEF monitoring is the current standard of care to monitor for cardiotoxicity, its accuracy, reproducibility, relevance, and timeliness in predicting cardiac dysfunction remain topics of debate [57, 59]. There are a number of ongoing clinical studies and registries assessing the cardiac monitoring techniques and biomarkers in patients with HER2+ breast cancer (Table 3).

**Table 3:** Ongoing trials of cardiotoxicity monitoring and management

Trial	Population	Cohorts	Outcomes/endpoints	Status
COBC (NCT02571894)	Women with newly diagnosed BC receiving (neo)adjuvant CT ± TRAS *N* ≈ 320	Standard oncological follow-up vs standard oncological follow-up plus surveillance and treatment for subclinical cardiotoxicity	• Primary: cumulative incidence of cardiotoxicity at 1-year post-CT • Secondary: number of cardiotoxic events at 5 and 10 years post-CT; overall survival, biomarker (hs-TnT and BNP) and imaging results, and quality of life result at 1, 5, and 10 years post-CT	Active, not recruiting; estimated primary completion February 2020
EMBRACE-MRI (NCT02306538)	Women with HER2+ early BC receiving TRAS + CT (no prior treatment with anthracycline) *N* ≈ 136	All patients undergo cardiac MRI pre-treatment, after anthracycline treatment, during TRAS treatment, and at the end of all therapy	• Primary: incidence of myocardial edema with or without conventionally defined cardiotoxicity • Secondary: incidence of myocardial edema with or without ≥ 5% LVEF decrease	Recruitment ongoing; estimated primary completion October 2019
CCT (NCT01173341)	Women with HER2+ BC receiving chemotherapy ± TRAS *N* ≈ 625	Patients will receive ECG and blood draw, by patient treatment group (TRAS only, CT only, TRAS + CT)	• Primary: incidence of cardiac dysfunction or signs or symptoms of heart failure • Secondary: Incidence of LVEF change	Recruitment ongoing, estimated primary completion April 2029
TITAN (NCT01621659)	Patients with BC or lymphoma scheduled to receive anthracyclines and/or TRAS *N* ≈ 282	Patients will receive usual care or regular assessment and treatment by a multidisciplinary team	• Primary: ECG change from baseline to 1 year • Secondary: serum biomarker change from baseline to 1 year	Active, not recruiting; estimated completion May 2019
EACVI/HFA COT Registry	BC patients undergoing treatment with an agent with a known potential of cardiac toxicity and cardiac monitoring	All patients are observed at baseline and for 12 months (5-year follow-up planned)	• Examine clinical, imaging, and treatment practices for anti-BC drug-related cardiotoxicity in Europe	Ongoing; established in 2015

*BC* breast cancer, *BNP* B-type natriuretic peptide, *CT* chemotherapy, *ECG* electrocardiogram, *HER2+* human epidermal growth factor receptor-2-positive, *LVEF* left ventricular ejection fraction, *MRI* magnetic resonance imaging, *TRAS* trastuzumab, *TnT* troponin T

Echocardiography and cardiac magnetic resonance (CMR) are the current standards for assessing LVEF due to ease of use, widespread availability, and lack of radiation [53]. Techniques to improve the reliability and reproducibility of echocardiography, such as 3-dimensional and contrast echocardiography, are under investigation [72, 73]. Three-dimensional echocardiography is preferable due to high reproducibility but may not be widely available and may also rely on high-quality images and a knowledgeable operator [53]. Contrast echocardiography provides more accurate estimations of ejection fraction and may be particularly useful in patients with a poor first reading by providing enhanced definition and improving interobserver reliability [53, 72]. Measurements of myocardial strain are an active area of research and have been demonstrated to be a reliable early detector of chemotherapy-induced cardiotoxicity [74]. Tissue Doppler imaging (TDI), used to measure systolic and diastolic velocity, may provide more sensitive measures of early changes predictive of cancer therapy-related heart failure, even in the absence of LVEF changes [75]. Although TDI should be considered, its use is limited by both availability and technical issues such as reproducibility [53, 75]. Techniques for the measurements of global longitudinal strain (GLS), such as speckle tracking echocardiography (STE), may also provide an easily quantifiable mechanism for early detection of cardiotoxicity before LVEF decrease is noted [74]. Further research is needed to determine whether the benefit of these enhanced cardiographic techniques warrants the additional cost over traditional 2-dimensional echocardiography. Regardless of the modality chosen, the universal recommendation is to use the same technique for baseline assessment and all subsequent follow-up assessments to obtain the most accurate comparisons.

Circulating biomarkers of cardiac damage may provide even earlier and more sensitive detection of potential cardiac dysfunction than imaging techniques. Troponin-1, a marker of cardiomyocyte injury that precedes decreased LVEF, is detected at elevated levels in patients receiving HER2-targeted therapy with chemotherapy for breast cancer [76, 77]. Using a combination of both troponin-1 levels and advanced imaging such as STE may provide early indications of LVEF decrease during trastuzumab treatment and may be beneficial for patients at high risk of cardiotoxicity [18, 52, 53]. Other biomarkers, including myeloperoxidase (MPO), C-reactive protein (CRP), and growth differentiation factor-15 (GDF-15) are under investigation for patients with cancer receiving potentially cardiotoxic therapy [76, 78]. The encouraging results of initial studies into biomarkers of cardiac injury warrant their further assessment in prospective clinical studies to determine optimal selection of measurement, timing of analysis, and appropriate intervention based on results [18, 53].

## Conclusions

The past several decades have seen a rapid increase in the number of agents available to treat HER2+ breast cancer. This has led to dramatic increases in disease-free survival for patients who undergo adjuvant therapy for early-stage disease and in longer lives for patients with metastatic disease (who thereby are often receiving treatment for prolonged periods). Although enormously beneficial in terms of disease control, these therapies are associated with some risks. The initial phase 3 trial of trastuzumab revealed an unexpected significant increase in cardiotoxicity. Additional HER2-targeted therapies, including lapatinib, T-DM1, pertuzumab, and others, also noted increases in cardiotoxic outcomes, albeit at less frequent rates. As these therapies are often administered in combination, or sequentially with other potentially cardiotoxic chemotherapy agents such as anthracyclines, special attention is necessary to manage and mitigate these potentially serious outcomes.

With years of experience in the clinical trials setting and in real-world practice, clinical practice guidelines have been developed for the optimal management of cardiotoxicity with HER2-targeted therapies. Routine cardiac monitoring, dose modification or cessation of anticancer therapies, and pharmacologic treatment of early cardiotoxicity all contribute to improved cardiac outcomes in patients with HER2+ breast cancer. However, for patients with pre-existing cardiac conditions or those who develop cardiotoxicity during the course of therapy, there remains an urgent need for effective treatments. A number of ongoing studies will provide additional information on improved monitoring techniques, interventions, and strategies for the optimal treatment of patients with cardiac disease at baseline. We eagerly await the results of such trials, which will help improve the treatment of HER2+ breast cancer in an expanded number of patients.

## Data Availability

Data sharing not applicable to this article as no datasets were generated or analyzed during the current study.

## Abbreviations

ACE: Angiotensin converting enzyme
AE: Adverse event
ASCO: American Society of Clinical Oncology
CHF: Congestive heart failure
CMR: Cardiac magnetic resonance
CRP: C-reactive protein
EGFR: Epidermal growth factor receptor
ESC: European Society of Cardiology
ESMO: European Society for Medical Oncology
GDF-15: Growth differentiation factor-15
GLS: Global longitudinal strain
HER2+: Human epidermal growth factor receptor-2-positive
LVEF: Left ventricular ejection fraction
MBC: Metastatic breast cancer
MPO: Myeloperoxidase
NCCN: National Comprehensive Cancer Network
STE: Speckle tracking echocardiography
TDI: Tissue Doppler imaging
T-DM1: Trastuzumab emtansine
TKI: Tyrosine kinase inhibitor
